# Fracture Analysis and Fatigue Strength Calculation of Anchor Bolt Used in Circulating Water Pump in Nuclear Power Plant

**DOI:** 10.1155/2021/7019861

**Published:** 2021-10-04

**Authors:** Qiang Chen, Shuai Zu, Yinhui Che, Dongxiong Feng, Yang Li

**Affiliations:** Suzhou Nuclear Power Research Institute Co., Ltd., Shenzhen 518120, China

## Abstract

A circulating water pump is a key equipment of cooling systems in nuclear power plants. Several anchor bolts were broken at the inlet rings of the same type of pumps. The bolts were turned by a special material for seawater corrosion protection. There were obvious turning tool marks at the root of the thread, which was considered as the source of the crack. The fatigue crack extended to the depth of the bolt, causing obvious radiation stripes on the fracture surface, which was a typical fatigue fracture. Obvious overtightening characteristics were found at the head of the broken bolt. Fracture and energy spectrum analysis showed that the bolt was not corroded. The axial vibration of the pump was measured. The static tensile stress along the bolt axis caused by the preload, the axial tensile stress caused by the axial vibration, and the torsional stress were calculated, respectively. According to the fatigue strength theory, the composite safety factor of the bolt fatigue strength was 1.37 when overtightening at 1.2 times the design torque, which was less than the allowable safety factor of 1.5-1.8, so the bolt was not safe, which further verified the conclusion of fracture analysis. The reason for the low safety factor was caused by the overtightening force. The improvement method was to control the bolt preload or increasing the bolt diameter.

## 1. Introduction

**Figure 1:**
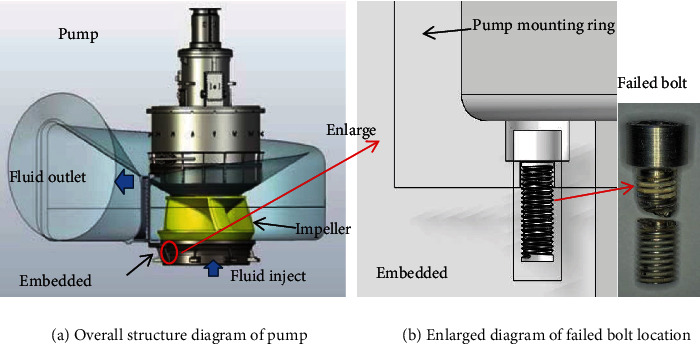
Structure of embedded parts under circulating water pump.

A cooling water pump is a very important equipment in nuclear power plants. During overhaul, it was found that the fixing bolts of the embedded parts of four CR1QS1 pumps were broken. The pump is a single-stage, vertical, bottom-suction concrete volute centrifugal pump. The pumps were fixed on the concrete embedded parts with 8 M12 × 1.75 hexagon socket bolts through the mouth ring, as shown in Figure 1. The purpose of the protective cap is to protect the bolt from erosion. The working medium of the pump is sea water.

The common failure modes of bolt fracture are fatigue fracture, stress corrosion cracking, and overload fracture. Due to the large stress concentration of a bolt thread, it is easy for a fatigue source to form at the root, and the possibility of fatigue fracture is high. The bolt fracture studied by González et al. occurred at the second turn of the screw thread, which was caused by hydrogen embrittlement [1]. The bolt studied by Shafiei and Kazempour-Liaisi had M23C6 carbide, which was the source of the fatigue crack. The crack propagates along the grain boundary, and finally, fatigue fracture occurs [2]. Li et al. found that surface decarburization of the bolts and stress concentration at the bolt thread neck decreased the fatigue strength [3]. Wu et al. studied the corrosion fracture mechanism of cable bolts [4]. The fracture had general fatigue fracture characteristics. There were corrosion fatigue crack sources and radial fatigue crack propagation traces. Hydrogen-assisted stress corrosion cracking was the main fracture mechanism of cable bolts failure. The fatigue crack source of the bolt-sphere joint was pitting caused by corrosion [5]. Wen et al. [6] studied the fracture of a 20MnTiB steel high-strength bolt. Microdefects were found near the bottom of the thread. Considerable stress and corrosion accelerated the crack propagation of the bolt. The working capacity of a rock bolt decreased by 25-50% when it worked under the condition of rock and groundwater corrosion [7].

It is generally believed that the fatigue strength of bolts is only related to the stress amplitude. The fatigue strength only studied the stress amplitude of bolt tensile stress [7–10]. For example, the bolt fatigue strength condition was that the allowable stress amplitude was equal to 90 MPa [8], and the fatigue curve studied was the Δ*σ* − *N* curve [9]. However, in practice, many examples showed that the failure of bolts was related to the average stress (i.e., bolt preload) [11, 12]. The reason for a bolt fracture was that the safety factor is insufficient due to excessive preload [11, 12]. The safety factor of static strength is obtained by preloading, the safety factor of variable stress is obtained by strain, and the safety factor is modified by Goodman's theory [13].

In this paper, the fracture analysis, mechanical property analysis, and energy spectrum analysis of the broken bolt are carried out. At the same time, the fatigue strength of the bolt is calculated, the failure causes are found out, and the improvement suggestions are put forward. Finally, the calculation method of the bolt fatigue strength is proposed.

## 2. Fracture Analysis of Bolt

### 2.1. Fracture Analysis

**Figure 2:**
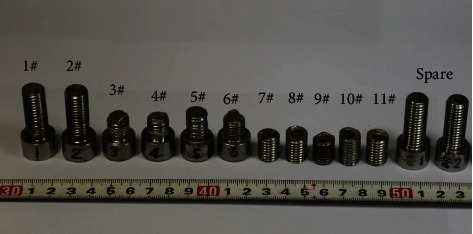
Bolts in service and spare parts.

The bolts in service are shown in Figure 2, in which Nos. 1 and 2 were the unbroken bolts, Nos. 3-6 were the head of the broken bolts, and Nos. 7-11 were the rest of the broken parts of the broken bolts. Compared with the spares, their surfaces were the same as the serviced bolts, indicating that there was no corrosion.

**Figure 3:**
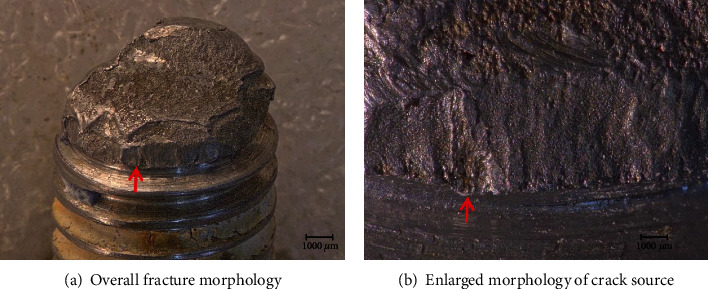
Fracture of No. 3 bolt in Figure 2.

The fracture of No. 3 bolt in Figure 2 is representative. Take it as an example to illustrate the fracture form of bolts. Figures 3(a) and 3(b) are the overall morphology and local morphology of the No. 3 bolt, respectively. There are obvious radial lines on the edge of the thread teeth, which is the fracture source as the point indicated by the arrow. The fracture source extends to the core, and then the bolt breaks when the crackle reaches the middle. This is the instantaneous fracture zone region, where the section is rough and uneven. The instantaneous breaking zone occupies a relatively large area, indicating that there is a large residual pretightening force when the bolt is broken.

**Figure 4:**
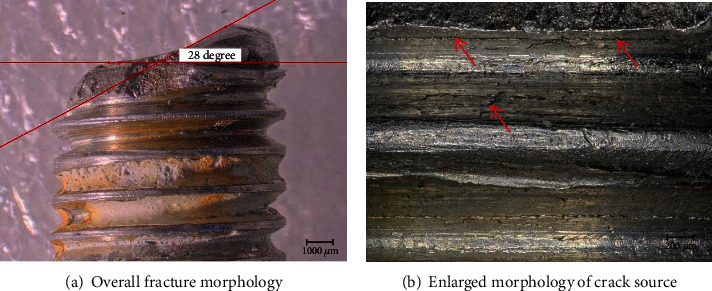
Macroscopic observation of thread profile of No. 3 bolt.

The macromorphology of No. 3 bolt fracture was observed by stereoscope, as shown in Figure 4(a). The fracture was uneven, with the thread teeth about 28°, which was about the direction of principal stress. Further zooming in and observing what is shown by the arrows in Figure 4(b), the source of the cracks is located at the machining tool mark at the root of thread, and there are a lot of microcracks around.

**Figure 5:**
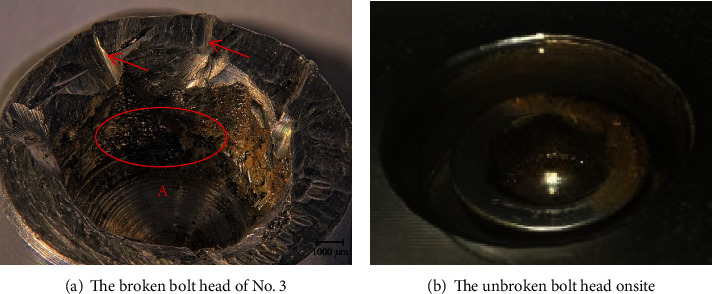
Comparative morphology of hexagon socket head.

Figure 5(a) is the morphology of the inner hexagon of the head of No. 3 broken bolt. The top of the bolt head is damaged when the sample was taken on site, as shown by the arrow. But the inner hexagon area is damaged during tightening, as shown in the region.  Figure 5(b) shows the morphology of the unbroken bolt head, with the inner hexagon of the screw head intact. The comparison shows that the broken bolts have overtightening behavior when they were installed.

**Figure 6:**
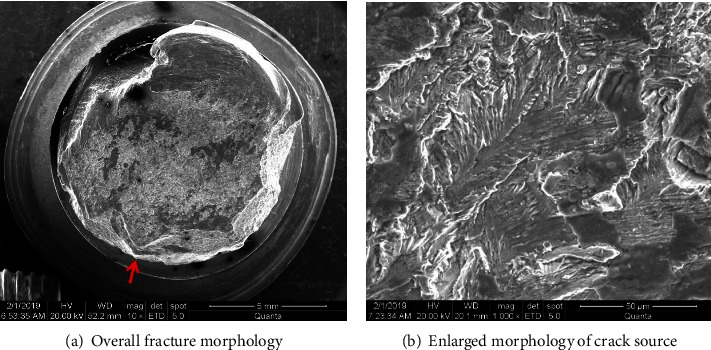
SEM photos of No. 3 bolt.

Figure 6(a) is the overall graph taken with a Scanning Electron Microscope (SEM), which shows the fracture source by arrow.  Figure 6(b) is a micrograph of the expansion zone, which shows typical fatigue fracture characteristics. This shows that the process of fracture propagation also has the effect of alternating stress.

**Figure 7:**
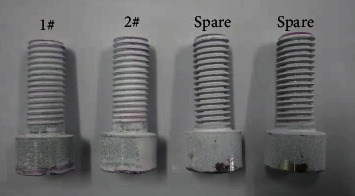
Macroimage of dye penetrant inspection for screws.

**Figure 8:**
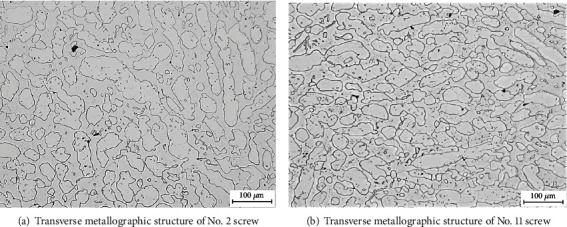
Microstructure of the sample bolts.

Figure 7 shows the macroimages of four unbroken screws through dye penetrant inspection, and no cracks are found on the surface. The metallographic structures of the unbroken and broken bolts are, respectively, shown in Figures 8(a) and 8(b), which show an austenite + ferrite structure. This conforms to the characteristics of dual phase steel, without obvious abnormality.

### 2.2. Research on Bolt Metallurgy

The bolts were made of a special material for seawater corrosion protection. Due to the small quantity, they were manufactured by turning. The chemical composition meets the ASTM s32760 standard, see Table 1. Using the XHB-3000 Digital Brinell Hardness Tester, the average hardness of bolts is 230-240 HBW, equivalent to grade 8.8 (Chinese national standard GB3098.1), which also meets the requirements of ASTM s32760 of less than 310 HBW.

Using an ONH836 hydrogen, oxygen, and nitrogen analyzer, the contents of gas elements N, H, and O were tested and shown in Table 2. The content of nitrogen meets the requirements of standard value, and the contents of hydrogen and oxygen are also low. In addition, the bolt did not have intergranular stress corrosion cracking, so the bolt fracture had nothing to do with the influence of gas content.

By the AG100KNG universal testing machine, the tensile properties of sample bolts were tested, as shown in Table 3. The results all meet the requirements of standard values, and the mechanical properties are normal. According to the empirical formula recommended in the mechanical design manual, the symmetrical cycle fatigue limit *σ*_−1_ and torque yield limit *τ*_*s*_ are estimated as follows:

(1) $$\begin{array}{c} \sigma_{-1}\approx 0.28\left(\sigma_{b}+\sigma_{s}\right)=0.28\times \left(814+569\right)=387\text{ MPa}, \end{array}$$

*τ*_*s*_ ≈ 0.58*σ*_*s*_ = 0.58 × 569 = 330 MPa.

### 2.3. Energy Disperse Spectroscopy Analysis

Table 4 shows the composition of the fracture surface after cleaning by Energy Disperse Spectroscopy (EDS). The result is the same as the previous conclusion in Section 2.1, that is, as can be seen in Figure 2, the broken bolts were as glossy as the spare parts, and there was obviously no corrosion.

## 3. Calculation of Bolt Fatigue Strength

### 3.1. Bolt Stress Analysis

#### 3.1.1. Pretightening Stress Calculation

The stress of a bolt includes the preload and the working load. There are two preloads: one is the axial tensile stress and the other is the torsional stress around the axis. The working load acts on the axial direction of the bolt, and the calculation method is shown in Section 3.1.2. This section mainly calculates the tensile stress *σ*_*m*_ and torsional stress *τ*_*m*_ caused by the preload.

The bolts should be tightened when they are installed; that is, they are subject to the preload (tension) and friction torque. When working, it may be subjected to the variable stress of axial tension. In this paper, the finite element method is used to calculate the tensile stress *σ*_*m*_ and torsional stress *τ*_*m*_ by ANSYS Workbench 15.0 software.

**Figure 9:**
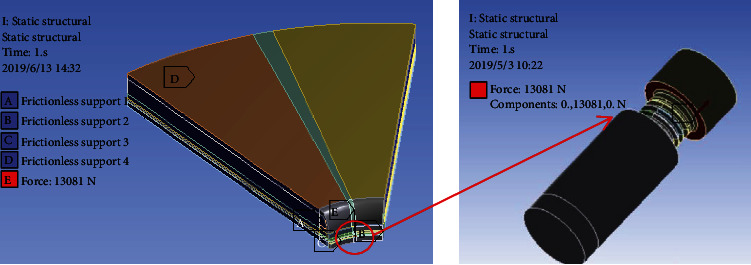
Finite element model.

The pump and the foundation ring are connected by 8 bolts. The finite element model takes 1 bolt and one eighth of the foundation including the ring and concrete, as shown in Figure 9. According to the equipment maintenance manual, the installation torque of the bolt is 40.5 Nm, the torque coefficient is 0.258, and the calculated preload is 13081 N.

**Figure 10:**
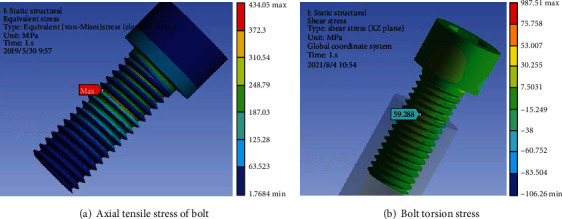
Stress value at design torque.

**Figure 11:**
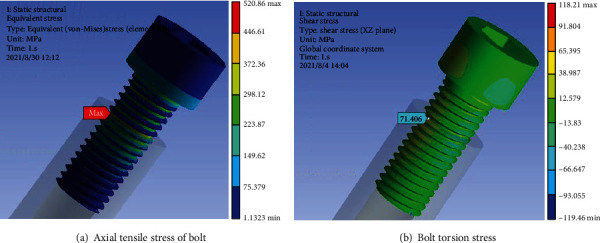
Stress value at 1.2 times design torque.

The axial tensile stress and torsional stress of the bolt are shown in Figures 10 and 11, respectively. The axial tensile stress *σ*_*m*_ is 434.05 MPa, and the torsional stress *τ*_*m*_ is 59.29 MPa at design torque. If the overtightening torque reaches 1.2 times the design value, the axial tensile stress *σ*_*m*_ is 520.86 MPa, and the torsional stress *τ*_*m*_ is 71.41 MPa. The inner hexagon of the broken bolt head has been seriously damaged, and the actual torque is far greater than 1.2 times the design value.

#### 3.1.2. Calculation of Bolt Working Stress

**Figure 12:**
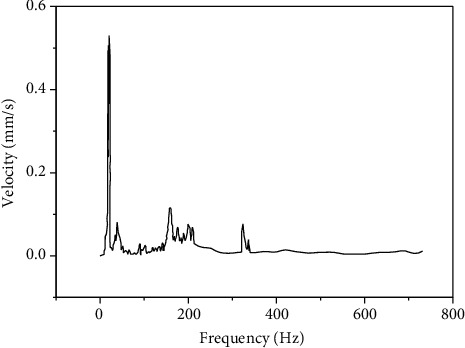
Vibration excitation load spectrum.

When the pump runs, the impeller will have a working load, acting on the bolt axis direction. The stress is a symmetrical cyclic strain produced by the axial vibration when the pump is running. The axial load was obtained by actual measurement. A speed sensor was installed at the bearing, and the excitation spectrum load was the relationship between the speed and the frequency spectrum, as shown in Figure 12.

**Figure 13:**
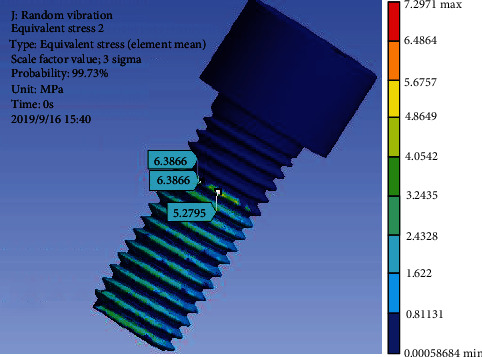
Equivalent stress of bolt.

The workbench random vibration analysis module was used to calculate the stress response of vibration fatigue. The excitation was loaded on the concrete foundation. The finite element equivalent stress diagram is shown in Figure 13, in which the maximum equivalent stress *σ*_*a*_ is 7.3 MPa.

### 3.2. Bolt Strength Calculation

The axial force of the bolt is similar to that of the shaft, so the formula of the safety factor of fatigue strength is as follows:

(2) $$\begin{array}{c} S_{\sigma }=\frac{\sigma_{-1}}{\left(k_{\sigma }\sigma_{a}/\varepsilon_{\sigma }\beta \right)+\psi_{\sigma }\sigma_{m}}, \end{array}$$

where *σ*_−1_ is the symmetrical fatigue limit of 387 MPa, as calculated by equation (1). *σ*_*a*_ is the working stress of 7.3 MPa, as calculated in Section 3.1.2. *σ*_*m*_ is the axial stress caused by the pretightening force, as calculated by FEM in Section 3.1.1. *k*_*σ*_ is the stress concentration factor, and take *k*_*σ*_ = 3 from the mechanical design manual because it was manufactured by turning. *ε*_*σ*_ is the size factor, and take 1 for the M12 bolt. *β* is the enhancement coefficient, and take 1 because of no enhancement. *ψ*_*σ*_ is the stress conversion factor, calculated as follows:

(3) $$\begin{array}{c} \psi_{\sigma }=\frac{\sigma_{-1}}{\sigma_{b}}=\frac{387}{814}=0.475. \end{array}$$

So, the safety factor of normal stress at design torque is as follows:

(4) $$\begin{array}{c} S_{\sigma }=\frac{\sigma_{-1}}{\left(k_{\sigma }\sigma_{a}/\varepsilon_{\sigma }\beta \right)+\psi_{\sigma }\sigma_{m}}=\frac{387}{\left(\left(3\times 7.3\right)/\left(1\times 1\right)\right)+0.475\times 434.05}=1.7. \end{array}$$

The safety factor of normal stress at 1.2 times the design torque is as follows:

(5) $$\begin{array}{c} S_{\sigma }=\frac{\sigma_{-1}}{\left(k_{\sigma }\sigma_{a}/\varepsilon_{\sigma }\beta \right)+\psi_{\sigma }\sigma_{m}}=\frac{387}{\left(\left(3\times 7.3\right)/\left(1\times 1\right)\right)+0.475\times 520.86}=1.44. \end{array}$$

The torsional stress produced by friction during preloading is static stress.

So, the safety factor at design torque is as follows:

(6) $$\begin{array}{c} S_{\tau }=\frac{\tau_{\text{s}}}{\tau_{0}}=\frac{330}{59.29}=5.57. \end{array}$$

The safety factor of normal stress at 1.2 times the design torque is as follows:

(7) $$\begin{array}{c} S_{\tau }=\frac{\tau_{\text{s}}}{\tau_{0}}=\frac{330}{71.41}=4.62. \end{array}$$

The composite safety factor at design torque is as follows:

(8) $$\begin{array}{c} S=\frac{S_{\sigma }S_{\tau }}{\sqrt{S_{\sigma }^{2}+S_{\tau }^{2}}}=\frac{1.7\times 5.57}{\sqrt{1.7^{2}+5.57^{2}}}=1.63\geq \left[S\right]=1.5\sim 1.8. \end{array}$$

The safety factor of normal stress at 1.2 times the design torque is as follows:

(9) $$\begin{array}{c} S=\frac{S_{\sigma }S_{\tau }}{\sqrt{S_{\sigma }^{2}+S_{\tau }^{2}}}=\frac{1.44\times 4.62}{\sqrt{1.44^{2}+4.62^{2}}}=1.37\leq \left[S\right]=1.5\sim 1.8. \end{array}$$

According to the mechanical design manual, the allowable safety factor is 1.5~1.8. The composite safety factor at design torque is greater than the lower allowable safety factor. However, when the overtightening torque reaches 1.2 times the design torque, the safety factor has been less than the limit, so the bolt is not safe. The reason for the low composite safety factor is that the safety factor of axial tension is too low, which is 1.44 only. There are two factors affecting the safety factor of axial tension. The first part is the working load, and the other is the preload. The stress caused by the working load is 7.3 MPa only, which is very small. Even if multiplied by the stress concentration factor of 3, 21.9 MPa, it is still very small and not enough to cause failure. However, the stress of 520.86 MPa caused by the preload is relatively large. Therefore, the measure to improve the safety factor is to control the bolt preload or increase the bolt diameter.

## 4. Conclusion

Through the above analysis of bolt fracture, metallurgy, and strength, the following conclusions can be drawn:

1. There are obvious crack sources at the root of the thread, and there is an obvious fatigue fracture zone and an instantaneous fracture zone at the cross section. The fatigue fracture zone is typically radial and has typical fatigue fracture characteristics
2. The bolt safety factor at 1.2 times the design torque is 1.37, which has been less than the allowable safety factor of 1.5-1.8. Therefore, the fatigue strength of bolts is insufficient, and a bolt fracture is due to fatigue failure when the bolt is overtightened
3. The failure of bolts is not caused by seawater corrosion. The surface of the broken bolt is bright, and there is no trace of corrosion
4. The key cause of a bolt fracture is too much preload. The measure to improve the safety factor is to control the bolt preload or increase the diameter of the bolt

## Figures and Tables

**Table 1:** Chemical composition of bolts (wt%).

C	Mn	P	S	Si	Cr	Ni	Mo	Cu	W
≤0.030	≤1.00	≤0.030	≤0.010	≤1.00	24.0-26.0	6.0-8.0	3.0-4.0	0.50-1.00	0.50-1.00

**Table 2:** Gas element test of bolts.

Bolt sample	Gas content (wt%)		
	N	H	O
No. 2	0.28	<0.00006	0.022
No. 10	0.28	0.0001	0.022
Standard value	0.20-0.30	—	—

**Table 3:** Test results of tensile properties of sample bolts at room temperature.

	Tensile strength *σ*_*b*_ (MPa)	Yield strength *σ*_*s*_ (MPa)	Elongation (%)	Reduction of area (%)
Mechanical property	814	569	38.5	70
ASTM S32760	≥750	≥550	≥25	—

**Table 4:** Composition of washed fracture surface.

Element	Weight (%)	Atomic (%)	Test position
C K	6.20	20.20	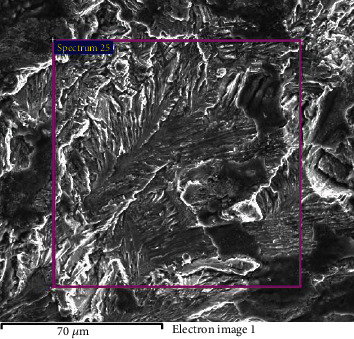
O K	7.39	18.09	
Al K	0.36	0.52	
Si K	0.85	1.18	
Cr K	22.41	16.88	
Mn K	1.18	0.84	
Fe K	54.28	38.06	
Ni K	4.80	3.20	
Mo L	2.53	1.03	
Totals	100.00	100.00	

## Data Availability

All data generated or analyzed during this study are included in this article.
